# Effects of Internet Cognitive Behavioral Therapy for Insomnia and Internet Sleep Hygiene Education on Sleep Quality and Executive Function Among Medical Students in Malaysia: Protocol for a Randomized Controlled Trial

**DOI:** 10.2196/59288

**Published:** 2024-12-11

**Authors:** Vijandran Mariappan, Firdaus Mukhtar

**Affiliations:** 1 Department of Psychiatry Faculty of Medicine and Health Sciences Universiti Putra Malaysia Serdang Malaysia

**Keywords:** sleep quality, cognitive behavioral therapy, sleep hygiene, medical students, executive function, Malaysia, insomnia

## Abstract

**Background:**

Medical students are frequently affected by poor sleep quality. Since poor sleep quality has negative physiological and psychological consequences such as on executive function, there is an opportunity to improve sleep quality and executive functions using non-pharmacological intervention such as cognitive behavioural therapy.

**Objective:**

The aim of this study therefore is to determine if improving sleep quality could improve executive functions in medical students with poor sleep quality by comparing cognitive behavioural therapy for insomnia (CBT-I) with sleep hygiene education (SHE) in a randomized controlled trial (RCT).

**Methods:**

A parallel group, RCT with a target sample of 120 medical students recruited from government-based medical universities in Malaysia. Eligible participants will be randomized to internet group CBT-I or internet group SHE in a 1:1 ratio. Assessments will be performed at baseline, post-intervention, 1 month, 3-months, and 6-months. The primary outcome is between-group differences in sleep quality and executive function post-baseline. The secondary outcomes include pre-sleep worry, attitude about sleep, sleep hygiene and sleep parameters.

**Results:**

This study received approval from the Research Ethics Committee in Universiti Putra Malaysia (JKEUPM-2023-1446) and Universiti Kebangsaan Malaysia (JEP-2024-669). The clinical trial was also registered in Australian New Zealand Clinical Trial Registry (ACTRN1264000243516). As of June 2024, the recruitment process is ongoing and a total of 48 and 49 students have been enrolled from the universities into the CBT-I and ISHE groups, respectively. All the participants provided signed and informed consent to participate in the study. Data collection has been completed for the baseline (pre-treatment assessment), and follow-up assessments for T1 and T2 for all the participants in both groups, while T3 and T4 assessments will be completed by July 2025. Data analysis will be performed by August 2025 and the research will be completed by December 2025.

**Conclusions:**

This study is the first attempt to design a CBT intervention to ameliorate poor sleep quality and its related negative effects among medical students. This research is also the first large-scale exploring the relationship between health status and CBT-mediated sleep improvement among medical students.

**Trial Registration:**

Australian New Zealand Clinical Trials Registry ACTRN12624000243516; https://www.anzctr.org.au/Trial/Registration/TrialReview.aspx?id=387030

**International Registered Report Identifier (IRRID):**

PRR1-10.2196/59288

## Introduction

### Background

It is scientifically acknowledged that sleep and executive functions undergo changes in prefrontal circuits that underlie executive functioning as part of healthy aging [1]. According to the model proposed by Harrison and Horne [2] and recent findings, selective sleep loss affects prefrontal cortex (PFC) circuitry, leading to changes in brain metabolism and impairing executive function in healthy adults [3-5]. Based on these findings, several studies have debated the vulnerability of the PFC to sleep loss [6,7]. A study investigating sleep problems and executive function among university students found a significant relationship between poor sleep quality (SQ) and impaired executive function [8]. Medical students, in particular, are frequently affected by poor SQ during their time in medical school [9].

Medical students from various countries have shown differing prevalence rates of poor SQ, including 44.2% in Nepal, 41% in Iran, 51% in the United States [10], 59% in Lithuania [11], 90% in China, 70.0% in Hong Kong, 32.5% in Nigeria, 37% in Saudi Arabia [12], and 62.0% in Ethiopia [13]. Recently, in Malaysia, a prevalence of 59.6% was reported among clinical students at International Islamic University Malaysia [14].

Sleep-related problems can result in substantial medical costs and human burden [15,16]. Sleep disorders such as insomnia affect approximately 25 million people in the United States annually, contributing to an estimated health care cost of US $100 billion [17]. Therefore, improving SQ among medical students through nonpharmacological interventions may help prevent or minimize the consequences of poor sleep, such as burnout.

To date, only a few effective nonpharmacological interventions are known to treat sleep-related issues such as poor SQ [18]. In a systematic review of nonpharmacological interventions aimed at improving sleep among university students (including 11% of medical students), cognitive behavioral therapy (CBT) accounted for 33.3% of the interventions, followed by sleep hygiene at 33.1%; relaxation, mindfulness, and hypnotherapy at 22.2%; and Gestalt therapy or imagery rehearsal therapy at 11.1%. The researchers concluded that CBT was effective in treating sleep disorders among university students. Additionally, other outcome variables, such as sleep duration, SQ, sleep onset latency (SOL), sleep hygiene, and dysfunctional beliefs about sleep, were examined [19]. Based on these findings, CBT and sleep hygiene education (SHE) were identified as the most commonly used interventions to improve SQ.

CBT for insomnia (CBT-I) is a nonpharmacological intervention recommended as the first line of treatment for improving SQ [20] and is considered the gold standard for insomnia treatment [21,22]. CBT-I was developed based on the Spielman behavioral model of insomnia [23] and is an extension of the stimulus control theory of insomnia [24]. It is a multicomponent psychotherapy, comprising elements such as sleep education, stimulus control therapy, sleep restriction therapy, cognitive therapy, sleep hygiene, and relaxation training [25,26].

CBT-I focuses on dysfunctional cognition and maladaptive behaviors that contribute to sleep problems. However, access to CBT-I is limited due to the shortage of trained personnel and the high cost of treatment [27-30]. Internet-based CBT-I (ICBT-I), with or without therapist guidance, has been successfully used for managing chronic insomnia [27,28]. Similarly, SHE has been reported to be equally effective in improving SQ among individuals aged 12 years and older [29]. However, SHE is a single educational intervention that focuses on sleep hygiene, whereas CBT-I is a multicomponent intervention. Therefore, CBT-I is considered a potentially more effective intervention for improving SQ and executive function among individuals, but evidence-based data to support this claim are lacking. Furthermore, studies comparing the effectiveness of CBT-I and SHE interventions are scarce. Despite recognizing SQ as a foundation of a healthy lifestyle, there is limited emphasis on sleep hygiene and sleep education in medical schools. Hence, this study protocol outlines the methodology of a randomized controlled trial (RCT) designed to examine the effectiveness of CBT-I and SHE on SQ and executive function among medical students. The secondary outcomes to be investigated include presleep worry, attitudes toward sleep, sleep hygiene, and sleep parameters.

### Conceptual Framework

**Figure 1:**
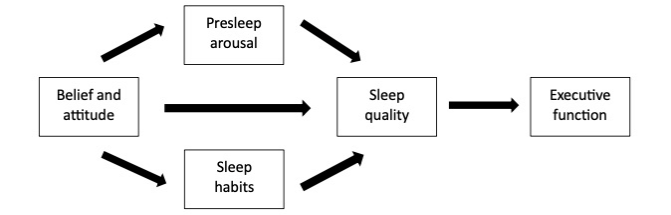
The cognitive model of insomnia.

This study will use several theories, namely, the cognitive models of insomnia, the hyperarousal models of insomnia, and the PFC circuitry model, to develop the theoretical framework for the interventions to be investigated. First, according to the cognitive model of insomnia (Figure 1), individuals with sleep problems often experience excessive worry, which prevents them from falling asleep and consequently leads to poor SQ [30]. As a result of this worry, individuals with sleep problems tend to focus on sleep-related internal threats (eg, bodily sensations) and external threats (eg, environmental noises). This selective focus causes them to become hyperaware of minor cues (previously unnoticed), which leads to excessive worry and anxiety. Additionally, individuals with sleep problems develop negative cognitions that further increase their cognitive and arousal activity, preventing them from falling asleep [30]. The model also suggests that individuals with sleep problems often engage in safety behaviors, such as napping or consuming alcohol, to relieve this arousal. However, these safety behaviors eventually worsen the worry and prevent individuals from challenging their negative sleep beliefs [30].

Second, the hyperarousal model of insomnia incorporates both the cognitive and behavioral models of insomnia, while integrating principles of classical conditioning [24,25]. This model posits that conditioned arousal is a key factor in perpetuating sleep problems. It further explains that repeated associations between poor sleep and the bed can increase conditioned arousal, with the bed and sleep environment becoming stimuli for heightened arousal [31]. Cognitively, individuals with increased sleep arousal begin to perceive “learned sleep–preventing associations,” thereby perpetuating their sleep problems [25].

Third, it is scientifically acknowledged that sleep and executive functions undergo alterations in prefrontal circuits that support executive function as part of healthy aging [1]. According to the model proposed by Harrison and Horne [2] and recent findings [3-5], selective sleep loss affects PFC circuitry, potentially leading to changes in brain metabolism that impact executive function in healthy adults. Building on these findings, several studies have debated the vulnerability of the PFC to sleep loss [6,7]. In this study, improvements in SQ may affect the PFC circuitry, particularly executive function. Consistent with this, our study aims to use ICBT-I to alleviate poor SQ and enhance executive function among medical students. The delivery of ICBT-I overcomes common limitations associated with traditional face-to-face administration, such as the lack of trained personnel, high costs, and limited treatment availability due to time constraints [30].

### Research Aim and Hypotheses

This study aims to elucidate the effects of improved SQ on executive function. The following hypotheses will be investigated in the trial:

- ICBT-I will lead to a significant improvement in SQ compared with the SHE intervention at 1 month (T2), 3 months (T3), and 6 months (T4) postintervention.
- ICBT-I will lead to a significant improvement in executive functioning compared with the SHE intervention at 1 month (T2), 3 months (T3), and 6 months (T4) postintervention.
- ICBT-I will lead to a significant improvement in total sleep time (TST), SOL, number of awakenings (NOA), wake time after sleep onset (WASO), and sleep efficiency (SE) compared with the SHE intervention at 1 month (T2), 3 months (T3), and 6 months (T4) postintervention.
- Dysfunctional beliefs, attitudes about sleep, presleep worry, and sleep hygiene will significantly mediate the between-group differences in study outcomes at 1 month (T2), 3 months (T3), and 6 months (T4) postintervention.

## Methods

### Trial Design

**Figure 2:**
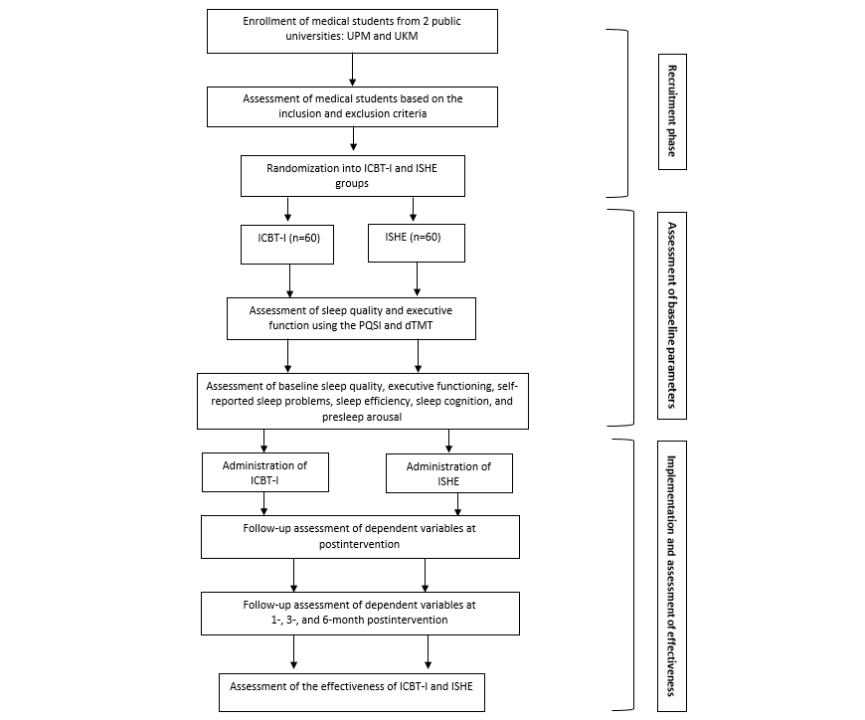
The trial design. dTMT: Digital Trail Making Test; ICBT-I: internet-based cognitive behavioral therapy for insomnia; ISHE: internet-based sleep hygiene education; PSQI: Pittsburgh Sleep Quality Index; UKM: Universiti Kebangsaan Malaysia; UPM: Universiti Putra Malaysia.

A 2-arm, parallel-group RCT will be conducted, comparing ICBT-I with internet-delivered SHE (ISHE). The trial design is summarized in Figure 2. The study population will consist of preclinical medical students who meet the established criteria for poor SQ, as indicated by self-report assessments, which are internationally recognized as standardized instruments. The medical students will be randomly assigned in a 1:1 ratio to either the ISHE or ICBT-I group. All interventions will be conducted online using a paid Zoom (Zoom Video Communications/Qumu Corporation) platform. As shown in Figure 2, participants will receive an electronic document containing the participant information statement, screening instruments, informed consent form, and randomized allocation to the intervention groups. Both baseline and follow-up assessments will be conducted either online or via mobile platforms. Participants will be able to print the participant information statement, and contact details (phone numbers and emails) will be provided for participants to reach the researchers before consenting and during the data collection process.

### Ethics and Dissemination

Ethical approval for this study was obtained from the Human Ethics Committees at Universiti Putra Malaysia (UPM; JKEUPM-2023-1446) and Universiti Kebangsaan Malaysia (UKM; JEP-2024-669). Informed consent will be obtained from each potential participant before their recruitment into the study. They will be briefed on their right to participate or decline, as well as their right to opt out of the study without penalty. Regarding privacy and confidentiality, data will be fully anonymized, and no detailed information about participants will be recorded. No incentives will be provided for participation in this study.

The RCT protocol aligns with the SPIRIT (Standard Protocol Items for Randomized Trials) guidelines [32], as shown in the recruitment and assessment procedures in Figure 2. The uploaded SPIRIT checklist can be found in Multimedia Appendix 1. Multimedia Appendix 2 presents the protocol approval obtained from the Australian New Zealand Clinical Trials Registry (ANZCTR; ACTRN12624000243516). Upon completion of the study, all data will be stored in the institutional database for a specified duration before being destroyed. The findings will be published as a research article in a journal, with no information that could reveal the participants’ identities.

### Sample Size Estimation

**Figure 3:**
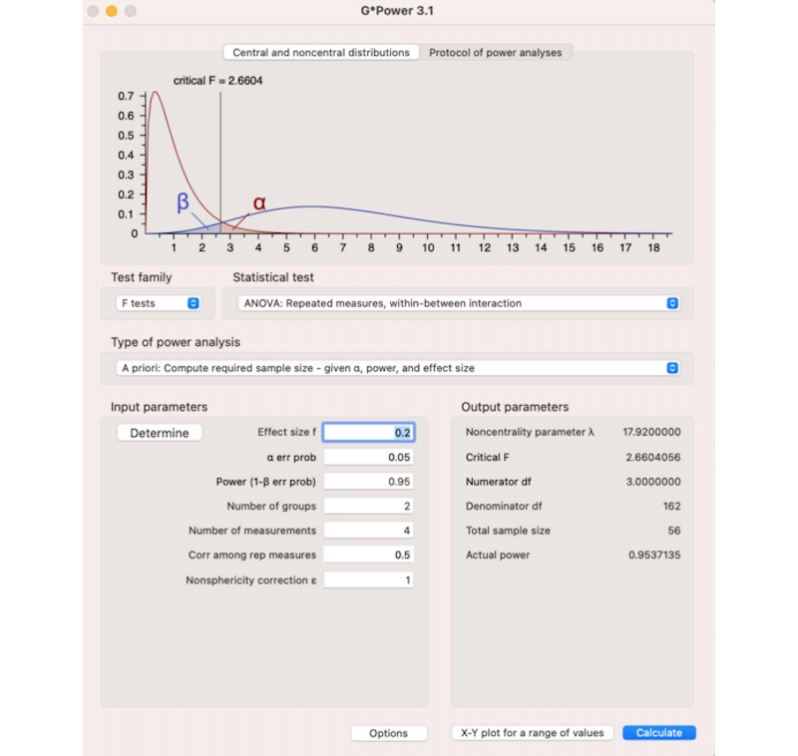
Statistical test used in computing the required sample size in G*Power software.

As shown in Figure 3, the sample size for the RCT was calculated using G*Power software (Heinrich-Heine-Universität Düsseldorf), selecting the *F* tests for analysis of variance: repeated measures, within-between interaction. The calculation was based on a precision level of 0.05, a study power of 95%, an effect size of 0.20, and a correlation coefficient of 0.5 among the outcome measures. Therefore, the minimum required sample size for each group is 56. A low effect size of 0.20 was chosen based on previous interventions addressing SQ [33]. After adjusting for a 10% dropout rate, the sample size was increased to 60 medical students per group. Given that this is a 2-arm RCT, a total of 120 medical students will be required to complete the study.

### Participants and Recruitment

The RCT will involve 120 medical students from 2 government-based medical universities: UPM and UKM. These institutions were selected for their convenience and proximity to the researchers. The inclusion of both universities is also intended to account for potential differences in medical curricula and academic schedules between government-based and private medical institutions. Participants will consist of volunteers, specifically undergraduate medical students in the preclinical years (ie, years 1-3). A list of potential participants will be compiled with permission from the dean. Recruitment advertisements will be posted across various platforms, including social media, campus spaces, and noticeboards, to facilitate participant engagement. Before the initial assessment, each participant will be required to complete the consent form and information sheet. Potential participants will undergo a screening process to assess their eligibility before being randomized into either the ISHE or ICBT-I group.

### Randomization and Allocation Concealment

As participants will be recruited from 2 different universities, a stratified randomization method will be used to ensure balance between the groups from each university. Eligible students will be grouped by university and gender before random selection. Gender will serve as a matching factor during the stratified randomization process to ensure a comparable number of female and male students are recruited from both universities.

Data will be collected from volunteer medical students, and the researchers will have no influence on the randomization process or access to future allocations. Each participant will be assigned an identification number and listed before being randomized into either the ISHE or ICBT-I group using online randomization software [34]. A total of 60 students will be allocated to each group.

### Study Location

The initial assessment and experiment will be conducted using a paid Zoom platform. Participants will be interviewed by the researcher using a semistructured questionnaire to determine their eligibility based on the inclusion and exclusion criteria before randomization.

### Eligibility

Upon expressing interest in participating in the RCT, medical students will be directed to a website with general information about the study and its procedures. They will then access the screening assessment document and consent form to complete the eligibility process. After providing informed consent and meeting the inclusion criteria, potential participants will be contacted by phone. During the call, the investigator will confirm the participant’s understanding of the RCT and the provisions for the internet-based interventions being investigated.

The inclusion criteria in Textbox 1 will be considered before recruiting a medical student for this study.

The criteria listed in Textbox 2 will be used to exclude individuals from this study.

**Study inclusion criteria.**

- Participants must be aged 18 years or older.
- Participants must currently be enrolled as medical students in Klang Valley, Selangor, Malaysia.
- Participants must be willing and able to provide written informed consent.
- Participants must be able to read and understand English.
- Participants must have access to reliable internet service, either at university or at home.
- Participants must be proficient in basic computer and internet operational skills, such as completing self-report questionnaires and online assessments.

**Exclusion criteria.**

1. Individuals currently suffering from a sleep disorder, such as restless legs syndrome or obstructive sleep apnea [35].
2. Individuals who have previously participated in sleep hygiene programs or undergone cognitive behavioral therapy for insomnia (CBT-I) treatment.
3. Individuals who currently consume alcohol.
4. Individuals with a history or present medical condition that may affect the use of CBT-I, such as recent cardiac surgery, epilepsy (self-reported) within the last 12 months, or currently experiencing an attack of multiple sclerosis.
5. Individuals with tight work schedules, such as night shifts, that may interfere with the completion of the online assessments.
6. Pregnant medical students.
7. Individuals unable to modify their sleep pattern or with limited opportunities to sleep as usual, such as those caring for an infant or having an infant at home.
8. Individuals currently being treated for insomnia and registered in 1 or more trial centers.
9. Individuals suffering from a severe physical health issue requiring surgery or with less than 6 months of prognosis for reassessment.
10. Individuals who have taken prescribed sleeping medication or pills for more than 2 nights in the last 14 days before recruitment into the study.
11. Individuals with suicidal intentions. No other mental or physical health problems will lead to exclusion from this study.

### Blinding

Single blinding will be implemented in this trial. As an online self-report assessment will be conducted, the researchers will remain blinded to the outcomes during the RCT. An automated email will inform the participants of the randomization results, so the medical students will know which group they are assigned to. Limited contact will be maintained between the researchers and participants to prevent any bias in the allocation process or influence on the subsequent assessments. The assessors will remain blinded even after participants contact the research team and reveal their allocation. A researcher (VM; University Putra Malaysia), who has access to all data, will perform the statistical analyses.

### Assessment Points

Participants in both groups will be assessed at baseline (week 0 or pretreatment assessment), week 6, 1 month, 3 months, and 6 months after the intervention. T0 represents the pretreatment assessment, while the subsequent 4 assessment points are designated as T1, T2, T3, and T4, respectively.

### Planned Interventions

#### The Internet Group (CBT-I)

A total of 6 modules focusing on the circadian and homeostatic mechanisms of human sleep regulation and their daytime consequences will comprise the CBT-I, as shown in Table 1. These modules outline the rationale for the key CBT-I interventions: (1) sleep restriction, which involves reducing time in bed to the average self-reported sleep duration from a 1-week sleep diary, plus 30 minutes, with a minimum of 6 hours of sleep and waking up at the same time daily, regardless of sleep duration; (2) stimulus control, which entails not going to bed unless feeling sleepy and not staying in bed unless asleep within 20 minutes; (3) discouraging napping; and (4) cognitive techniques, which involve restructuring dysfunctional beliefs, such as those about the consequences of sleep loss, and targeting unrealistic sleep perceptions or beliefs. A 10-minute video will be used to present the information.

**Table 1:** The structure and contents of the interventions.

Module	ICBT-I^a^ content	ISHE^b^ content
Module 1	Medical students will be introduced to the structure of the intervention and motivation for the therapy. Informing the participants that the treatment of choice for poor sleep quality and sleep disorders is the ICBT-I. Psychoeducation: information about sleep hygiene and regulation.	Medical students will be introduced to the structure of the intervention and motivation for the therapy. Informing the participants that the treatment of choice for poor sleep quality and sleep disorders is the ISHE. Psychoeducation: information about sleep hygiene and regulation.
Module 2	Sleep restriction: reducing time spent in bed, specifically to a minimum of 6 hours, in a stepwise manner. Using the sleep diary completed by the participants since the module to improve sleep pressure. Participants can increase the time spent in bed by 15 minutes if their sleep efficiency is greater than 85%.	Sleep hygiene I (sound, light, and temperature)
Module 3	Sleep hygiene and stimulus control: information on sleep-wake-rhythm, sleep-inhibiting substances, evening rituals, and physical exercise, as well as information on stimulus-control principles aimed at reassociating the bed with sleep.	Sleep hygiene II (food, exercise, and nicotine/alcohol/caffeine)
Module 4	PMR^c^ based on Jacobson [36] with downloadable written instructions and audio.	PMR based on Jacobson with downloadable written instructions and audio tape.
Module 5	Cognitive restructuring: identifying, reexamining, and modifying dysfunctional thoughts and beliefs about sleep and the consequences of poor sleep quality.	Stress management and sleep.
Module 6	Relapse prevention: actions to be taken to prevent poor sleep quality or relapse in sleep perturbations, including when it is appropriate to seek medical assistance and identify the conditions of safe pharmacotherapy.	Treatment evaluation and maintenance.

^a^ICBT-I: internet-based cognitive behavioral therapy for insomnia.

^b^ISHE: internet-delivered sleep hygiene education.

^c^PMR: progressive muscle relaxation.

The program includes a sleep diary in which medical students record their wake time, bedtime, sleep latency, night awakenings, and TST. Graphs will be generated based on the data entered by participants into the sleep diary. Additionally, various exercises will be included to ensure participants practice what they have learned. Participants will have the opportunity to repeat, internalize, and apply the modules, exercises, and examples to their daily lives at their own pace. Behavioral instructions will be provided to guide participants in completing the instruments, sleep diary, and responses during the program’s interactive phase.

Based on the preferred wake-up time and the average TST from the previous week, a sleep window will be proposed. To avoid excessive tiredness, a more flexible minimum time in bed of 6 hours has been chosen for this study. Participants are encouraged to extend their sleep window gradually. Once the sleep diary indicates an SE of 85% or greater over the past week, participants will be advised to add 15 minutes to their sleep window. However, if the recommended SE of 85% is not achieved, participants will be advised to reduce their sleep window based on their preferred average total sleep and wake-up times during the observation period. For those with an SE between 80% and 85%, the sleep window will remain unchanged, but it can be extended by 15 minutes if the efficiency is lower than 85%.

Besides the overall recommendations on sleep hygiene, the medical students will be encouraged to focus on specific behaviors that received the highest scores in the Sleep Hygiene Index (SHI; ie, reducing coffee or alcohol consumption just before bedtime if the participant reports frequent use). They will also be guided by the Dysfunctional Beliefs and Attitudes About Sleep-16 (DBAS-16), particularly for participants who score highly on the item “feel irritable, depressed, or anxious during the day, mostly because I did not sleep well the night before.” This reflects a strong belief in the negative impacts of poor SQ. Such participants will be advised to explore whether there are any underlying reasons for their anxiety and depression, aside from insomnia.

The internet program will serve as the primary medium for delivering all intervention materials, with participants engaging with the materials independently. They will also have the opportunity to consult a clinical psychologist (see the “Withdrawal and Crisis Management Procedure” section). Additionally, participants are strongly encouraged to use the feedback form to contact a specialist, particularly if they are newly recruited into the RCT, experiencing negative effects from the ICBT-I or ISHE interventions, or encountering methodological or technical difficulties with the program. The content of the intervention is entirely based on the well-established ICBT-I, which has been validated and tested in previous studies [37].

Each module will be completed with a homework report, which participants will fill out and submit to their therapists. The therapist will review the report, worksheets, and sleep diary data. Written feedback will be provided within 48 hours, and the therapist will grant the medical student access to the subsequent module. Meanwhile, if the participant is inactive for 1 week, the therapist will be alerted via a mobile phone SMS text message, serving as a reminder and encouragement. If necessary, another SMS text message will be sent, followed by emails, with phone calls as the final step. The therapist (FM) in this study is a licensed clinical psychologist with expertise in poor SQ and insomnia-related CBT.

#### The Internet-Based SHE

The ISHE modules provide psychoeducation on sleep, insomnia, and goal setting for treatment. The modules cover various topics including sleep hygiene I (light, sound, and temperature), sleep hygiene II (exercise, food, and alcohol/nicotine/caffeine), stress management, applied relaxation, mindfulness, and treatment evaluation and maintenance.

At the end of each module, participants will use a homework report checklist to review their assignments. The research team recommends daily entries in the sleep diary, encouraging participants to explore factors and patterns that affect their sleep. Participants are also encouraged to write comments in the discussion forum for each module and submit a post on a specific theme. While some content in the ISHE overlaps with the CBT-I, it does not include components such as stimulus control, sleep restriction, and cognitive restructuring.

### Treatment Adherence

Treatment adherence will be ensured through a standardized approach to delivering the intervention. The treatment manual outlines the key therapeutic information provided to the medical students, minimizing the influence of individual therapists. Additionally, the use of a therapist manual and supervision will further support adherence to the treatment in both groups.

### Outcome Measures

#### Overview

An email will be sent to participants requesting them to complete the online assessment in a secure online environment [38]. A consistent order of assessments will be followed by each participant at all time points. Participants who fail to complete the outcome measures within 2 days will receive follow-up email reminders. The research instruments consist of 56 items, including the Digital Trail Making Test (dTMT), and should take approximately 20-30 minutes to complete. A recent study demonstrated that online databases can effectively be used for data collection [37]. Participants’ demographic characteristics will be collected only at baseline, including descriptive data on alcohol use and smoking.

#### The Pittsburgh Sleep Quality Index

The Pittsburgh Sleep Quality Index (PSQI) is an instrument used to assess sleep over the previous month, and it is completed at the beginning of 2 study periods. This validated tool, based on polysomnographic research, consists of 19 self-reported items that evaluate 7 components: subjective SQ, sleep duration, sleep disturbance, sleep latency, habitual SE, use of sleeping medications, and issues related to sleep dysfunction. The instrument provides an index score ranging from 0 to 21 points. Scores greater than 5 indicate significant sleep problems, while lower scores reflect high or acceptable SQ [39].

#### Digital Trail Making Test

Executive functioning will be assessed using a self-reported online version of the dTMT, which includes TMT-A and TMT-B. In TMT-A, participants are required to connect a sequence of numbers in order. In TMT-B, participants must alternately connect numbers enclosed in circles and squares, following the sequence while ensuring that the pen remains in contact with the paper throughout the process. Participants’ executive functioning will be evaluated based on the time taken to complete the entire test and the accuracy of the number connections [40].

In this study, the dTMT will be used to assess medical students’ executive functioning. The TMTs are widely used in neuropsychological assessments due to their sensitivity to various neurological disorders. The test consists of 2 conditions (A and B) [41]. In part A, medical students will be instructed to draw a line sequentially connecting a total of 26 numbered circles as quickly as possible. In part B, each student will be asked to connect a series of 26 circles containing either a number or a letter, alternating between them. Both conditions will be scored based on the total time taken to complete the tasks and the frequency of errors made.

#### Other Sleep Measures

Average sleep parameters, including SOL, TST, NOA, WASO, SQ, and SE (calculated as TST/time in bed × 100), will be derived using the PSQI [42]. Inclusion criteria will be assessed using the baseline sleep diary. Participants will be instructed to document information about their SOL, WASO, NOA, early morning awakenings, number of nocturnal awakenings, TST, and SE for 10 days before the intervention (out of the previous 14 consecutive days) [43].

#### Dysfunctional Beliefs and Attitudes About Sleep-16

The DBAS-16 contains 16 items designed to measure sleep-related beliefs and attitudes [44]. A 10-point Likert scale is used to rate the items, ranging from 1=strongly disagree to 10=strongly agree. The minimum and maximum mean scores for the variables range from 1 to 10. Higher scores indicate a greater endorsement of dysfunctional beliefs about sleep. A previous study demonstrated that the instrument has an acceptable test-retest reliability of 0.83 and an internal consistency of 0.79, indicating its suitability for measuring dysfunctional beliefs and attitudes about sleep [45].

#### Pre-Sleep Arousal Scale

Sleep arousal among medical students will be assessed using the Pre-Sleep Arousal Scale (PSAS), which consists of 8 items each for measuring somatic and cognitive arousal [46]. A 5-point Likert scale is used to present items depicting symptoms of arousal at bedtime [46,47]. Higher scores reflect higher presleep arousal. The minimum and maximum scores for both types of arousal range from 8 to 40. The instrument demonstrates internal consistency, with Cronbach α of 0.82 for the cognitive scale and 0.79 for the somatic scale [47].

#### Sleep Hygiene Index

The SHI is a 13-item self-report instrument used to assess behaviors that may compromise sleep hygiene [48]. Poor sleep hygiene has been significantly associated with poor SQ [49-51]. Scores range from 0 to 52, with higher scores indicating behaviors that compromise sleep. The scores can also be analyzed using percentile ranks, where scores below 26 indicate good sleep hygiene, scores between 27 and 34 represent normal sleep hygiene, and scores of 35 and above indicate poor sleep hygiene [48]. The SHI has an internal consistency reliability coefficient of 0.66 [48].

### Assessment of Participants’ Safety

The risk of severe or serious adverse events in this study is low, as no such events have been reported in previous studies using CBT-I for the management of insomnia [49].

Nevertheless, studies have shown that sleep restriction therapy, one of the components of CBT-I, may increase levels of vigilance impairment and daytime sleepiness among participants, potentially leading to adverse situations such as suicide attempts, incidents, and formal complaints about the online intervention [49]. As both the intervention and assessment will be conducted online, the researcher may not be made aware of all such events. The researcher will, however, instruct participants to complete a feedback form to report any self-reported adverse effects [50].

### Withdrawal and Crisis Management Procedure

Participants can withdraw from the RCT at any time without penalty and are not required to provide reasons for their decision. In terms of crisis management, emergency psychiatric services will be available at UPM Teaching Hospital to assist participants in cases of crisis or distress that exacerbate their symptoms or lead to significant deterioration in functioning. All clinic attendees will have access to this service. The investigators will be notified if any medical student utilizes the emergency service, at which point a decision will be made regarding the RCT and whether the crisis qualifies as an adverse event related to the trial.

### Data Collection Procedures

The primary researcher (VM) will collect all data under the supervision of a registered and licensed clinical psychologist (FM). The researcher holds a master’s degree in clinical psychology and meets the minimum qualifications to administer all questionnaires to participants. Online assessments will be conducted by the clinical psychologist, using an instrument with Yes or No responses to items related to the inclusion and exclusion criteria. All clinical interviews will be audio-recorded to enable subsequent reliability assessments (see Multimedia Appendices 3 and 4 for the study information sheet and consent form, respectively). Participants who are willing to participate will be provided with a consent form. Their consent will be confirmed once they sign the form. Participants who meet the inclusion criteria and have PSQI scores greater than 5 will be grouped and asked to complete the online Trail Making Test, DBAS-16, PSAS, and SHI.

### Monitoring of Intervention and Follow-Up Assessment

The flowchart for the research activities is summarized in Figure 2. An SMS text message reminder will be sent by the Zoom administrator 1 day prior to and 30 minutes before the intervention. Follow-up assessments will be conducted at 1-month, 3-month, and 6-month intervals after the postintervention to reassess participants’ SQ, executive functioning, self-reported sleep problems, SE, sleep cognition, and presleep arousal. The same instruments described in the previous section will be used for the assessments. All participants will be monitored for adherence to the intervention, and potential dropouts will be identified and addressed accordingly.

### Data Analysis

Data will be analyzed using SPSS version 29 (IBM Corp.). Descriptive statistics will be used to obtain information on dropout rates, recruitment, and completeness. All participants will be included in the intention-to-treat analysis for assessing the main efficacy of the intervention, with no planned interim analysis for efficacy or futility. The baseline characteristics of participants will be presented according to their randomized group, with no inferential statistical tests performed at this stage.

For the RCT design, outcome measures will be assessed for normality and presented as either means with SD or medians with IQR, depending on the data distribution. Participants’ characteristics will be summarized using descriptive statistics, with categorical data presented as frequencies and percentages, and continuous data presented as means (SD) or medians (IQR). Normality will be tested using the Kolmogorov-Smirnov and Shapiro-Wilk tests.

Changes in all outcome measures (ie, SOL, TST, NOA, WASO, SQ, SE, dysfunctional beliefs about sleep, presleep arousal, and SHI) from pretreatment (T0) to the first (T1), second (T2), and third (T3) time points will be assessed for treatment effects using repeated measures analysis of variance. Both treatment and time interaction effects will be examined. Paired samples *t* tests will then be conducted within each condition to evaluate possible simple or main effects. Significant findings and statistical trends will be further analyzed using Cohen *d* effect size, which accounts for the correlation between pre- and posttreatment values.

Potential confounding factors, particularly class year and gender, will be addressed and analyzed as variables that may influence the research outcomes. These factors will be incorporated into the analysis through both univariate and multivariate linear regression analyses, allowing for the isolation of each factor and its association with the outcomes of interest.

Regarding the fourth hypothesis, mediation analyses will be conducted to explore whether changes in the outcome measures from pretreatment to posttreatment are significantly mediated by dysfunctional beliefs and attitudes about sleep, sleep hygiene, and presleep worry. The analysis will follow the traditional mediation model, where the direct, indirect, and total effects are defined in terms of the linear regression coefficients [51]. For example, when the mediation effect size is medium (ie, SM) and the intraclass correlation coefficient is 0.2, with 4 repeated measures, the Sobel method would require 191 participants to achieve 80% power. By contrast, the product-of-coefficients and bootstrap methods would require 188 and 185 participants, respectively, to achieve the same power [52]. This study will adopt the bootstrap method for mediation analysis due to its flexibility in terms of sample size requirements.

### Dissemination Policy

The results of this trial will be published in academic journals and presented at international and national conferences or clinical scientific meetings. Depending on the journal’s policies, the results will be made accessible online to interested readers.

## Results

Ethical approval for this research has been granted by the Ethics Approval Committee of UPM and UKM. As of June 20, 2024, the recruitment process is ongoing, with 48 students enrolled in the CBT-I group and 49 students in the ISHE group. All participants have provided signed informed consent to participate in the study. Data collection has been completed for the baseline (pretreatment assessment) and follow-up assessments at T1 and T2 for all participants in both groups. The T3 and T4 assessments will be completed by July 2025. Data analysis is scheduled for completion by August 2025, and the research is expected to conclude by December 2025.

## Discussion

### Expected Findings and Implications

CBT has long been established as the first-line treatment for individuals suffering from chronic insomnia, demonstrating positive effects and sustained improvements in sleep-related outcomes, as measured by both sleep diaries and objective indices of SQ [19-21]. A recent RCT with a well-defined placebo-controlled group found that dCBT produced effect sizes comparable to those of traditional face-to-face therapy [53]. However, it remains unclear whether CBT-I has a significant causal relationship with improving executive function among medical students compared with an SHE intervention. This knowledge gap has persisted for some time, and an investigation among medical students is therefore necessary. This study represents the first attempt to specifically examine SQ and executive function by comparing 2 established sleep interventions in this vulnerable group. The findings will be relevant to the care of medical students experiencing persistent SQ issues, particularly during their preclinical years. In particular, the results may highlight the potential for improving SQ as a novel therapeutic target, with benefits extending to generalized health outcomes and enhanced executive function. Furthermore, as an ICBT-I approach will be used in this trial, the study will demonstrate that addressing SQ among medical students is measurable, feasible, and effective.

Given the negative psychological and physiological consequences of poor SQ, such as burnout, suicidal ideation, and reduced empathy toward patients, this trial provides an opportunity to explore a nonpharmacological intervention for improving SQ and executive function among medical students. The findings will help bridge the important research gap identified in previous studies on CBT-I across diverse populations. These studies rarely focus on specific groups such as health professionals or young adults (eg, medical students). If poor SQ causally alters executive function, it is plausible to hypothesize that (1) CBT-I will improve both SQ and executive functioning and (2) the pathway through improved SQ will facilitate the treatment effects on executive functioning [54]. Furthermore, this trial provides a unique opportunity to expand existing knowledge, as only a few RCTs have investigated the roles of hyperarousal mediators (eg, presleep worry), cognitive mediators (eg, dysfunctional beliefs and attitudes about sleep), and behavioral mediators (eg, sleep hygiene) in CBT-I [20].

### Strengths and Limitations

This study will be the first RCT in Malaysia to investigate SQ and executive function using a dTMT among medical students. Additionally, it is the first to examine potential mediators, such as presleep worry and sleep hygiene, in the relationship between beliefs about sleep and SQ in this population. A representative sample will be recruited through a simple random sampling technique, allowing the findings to be extrapolated to public medical students in Malaysia and potentially other countries with similar educational demographics.

Nevertheless, this study has important limitations. The sample will be drawn only from medical students at UPM and UKM, meaning it may not be representative of all government-based undergraduate medical students in Malaysia. Additionally, the risk of response biases cannot be ruled out, particularly regarding subjective sleep experiences, which may influence the findings. Participants may also be inclined to provide socially desirable answers. Studies on Asian cultures have shown that, due to the cultural emphasis on modesty, Asians are likely to suppress the expression of positive emotions, which could impact the accuracy of self-reported data [55]. This study will use only a quantitative research approach, which may limit a comprehensive understanding of medical students’ perspectives and views on poor SQ symptoms and the strategies they use to address the issue. Additionally, there is a risk that participants may provide socially desirable answers when responding to the research instruments.

### Conclusions

This study is the first attempt to design a CBT intervention aimed at ameliorating poor SQ and its associated negative effects among medical students. It is also the first study to explore the relationship between improvements in SQ and executive function among medical students using CBT-I.

CBT-I focuses on addressing dysfunctional cognition and maladaptive behaviors that contribute to sleep problems. However, access to CBT-I is limited due to a shortage of trained professionals and the high cost of treatment [27-30]. ICBT-I, with or without therapist guidance, has proven effective in managing chronic insomnia [27,28]. Similarly, SHE has also been shown to improve SQ among individuals aged 12 years and older [29]. However, SHE is a single educational intervention that focuses on sleep hygiene, whereas CBT-I is a multicomponent intervention. Therefore, CBT-I is considered a potentially more effective intervention for improving SQ and executive function. However, evidence-based data to support this claim are lacking. Additionally, studies comparing the effectiveness of CBT-I and SHE are scarce. Despite recognizing SQ as a foundation of a healthy lifestyle, there is limited emphasis on sleep hygiene and sleep education in medical schools. Consequently, this study protocol outlines the methodology of an RCT aimed at evaluating the effectiveness of CBT-I and SHE on SQ and executive function among medical students.

## Appendix

Multimedia Appendix 1 SPIRIT (Standard Protocol Items for Randomized Trials) checklist.

Multimedia Appendix 2 Protocol registration and approval.

Multimedia Appendix 3 Study information sheet.

Multimedia Appendix 4 Study consent form.

## Abbreviations

ANZCTR: Australian New Zealand Clinical Trials Registry
CBT: cognitive behavioral therapy
CBT-I: cognitive behavioral therapy for insomnia
DBAS-16: 16-item Dysfunctional Beliefs and Attitudes
dTMT: Digital Trail Making Test
ICBT-I: internet-based cognitive behavioral therapy for insomnia
ISHE: internet-based sleep hygiene education
NOA: number of awakenings
PSAS: Pre-Sleep Arousal Scale
PSQI: Pittsburgh Sleep Quality Index
RCT: randomized controlled trial
SE: sleep efficiency
SHE: sleep hygiene education
SOL: sleep onset latency
SPIRIT: Standard Protocol Items for Randomized Trials
SQ: sleep quality
TST: total sleep time
UKM: Universiti Kebangsaan Malaysia
UPM: Universiti Putra Malaysia
WASO: wake time after sleep onset

## Data Availability

Important data underlying the results will be described in the manuscript once it is accepted for publication, with additional data provided in a supporting document.
