# Supplementary figures and images for: Effects of Internet Cognitive Behavioral Therapy for Insomnia and Internet Sleep Hygiene Education on Sleep Quality and Executive Function Among Medical Students in Malaysia: Protocol for a Randomized Controlled Trial
Source: JMIR Res Protoc. 2024 Dec 11;13:e59288. doi: 10.2196/59288 (PMC11669887; doi:10.2196/59288)

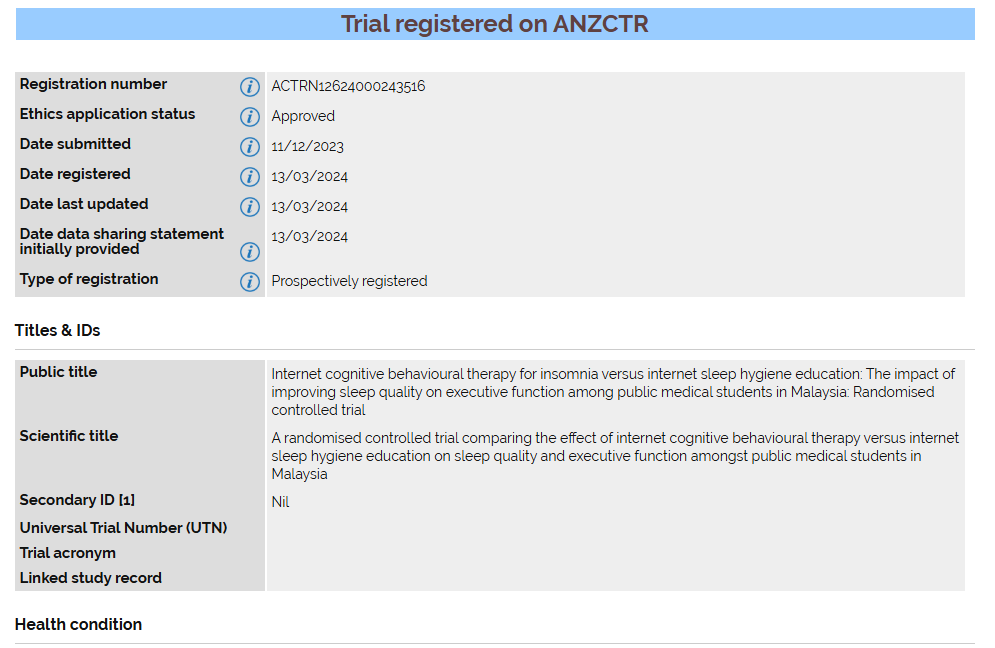

Supplement: Multimedia Appendix 2 [file resprot_v13i1e59288_app2.png]
