# Supplementary material for: Effects of Internet Cognitive Behavioral Therapy for Insomnia and Internet Sleep Hygiene Education on Sleep Quality and Executive Function Among Medical Students in Malaysia: Protocol for a Randomized Controlled Trial
Source: JMIR Res Protoc. 2024 Dec 11;13:e59288. doi: 10.2196/59288 (PMC11669887; doi:10.2196/59288)
Supplement: Multimedia Appendix 3 [file resprot_v13i1e59288_app3.docx]

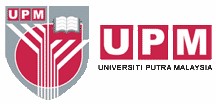
**JAWATANKUASA ETIKA UNIVERSITI UNTUK PENYELIDIKAN**

**MELIBATKAN MANUSIA (JKEUPM)**

**UNIVERSITI PUTRA MALAYSIA, 43400 UPM SERDANG, SELANGOR, MALAYSIA**

**RESPONDENT’S INFORMATION SHEET**

**STUDY TITLE :**

The Effectiveness of The Effectiveness of Group Internet Cognitive Behavior Therapy for Insomnia for Medical Students

**INTRODUCTION:**

The purpose of the study is to investigate the effectiveness of two methods of intervention in improving sleep quality and executive functioning. The two methods of intervention are (a) Internet Group Cognitive Behavior Therapy for Insomnia, (b) Internet Group Sleep Hygiene Education where participants with do regular procedures in terms of medical treatments. Respondents will be divided randomly into these two interventions and need to committedly involve this study for six months, followed by follow-up sessions (after one-month, three-months, and after six-months from post intervention). Follow-up sessions just in terms of assessments and not in the form of therapy. Psychological measurements will be conducted before and after interventions.

**WHAT WILL YOU HAVE TO DO?**

There are several tasks that you will have to do inthis study:

1. Socio-demographic profile- You are requested to complete the socio-demographic profile in the questionnare, including your contact details such as e-mail address and telephone numbers. This is very important in order for us to share with your the findings of the research after the study is completed.

1. Psychological measures_ Six sets of questionnaires will be distributed for you to answer throughout the study. One set of the questionnare will be distributed before the intervention, another one set will distributed after you have completed session four in the therapy and another four sets will be distributed after the intervention (i.e., immediately after the intervention being completed, after 1-month, 3-months, and after 6-months). The measures included in the questionnares are the Pittsburgh Sleep Quality Index (PSQI), online Trail Making Tests (dTMTs), SLEEP-50, Dysfunctional Beliefs and Attitudes about Sleep-16 (DBAS-16), Presleep Arousal Scale (PSAS), and Sleep Hygiene Index (SHI). All of the quationnares are in English language. You wil be remunerated with RM 50 for your effort upon completion of the questionnaire at the 6-month follow-up session.

1. Internet Group Cognitive Behavior Therapy for Insomnia (IGCBT-I): Group ICBT-I will help you understand about sleep quality and how to use cognitive and behavioral strategies to cope with sleep quality and executive functioning. You will be committed to six weeks programme of Group ICBT-I with six online sessions, once weekly frequency for 60 minutes.

1. Internet Group Sleep Hygiene Education (ISHE): Group ISHE will expose you to various techniques of sleep hygiene in order to improve sleep quality and executive functioning. You will be committed to six weeks programme of Group ICBT-I with six online sessions, once weekly frequency for 60 minutes.

**WHY YOU ARE BEING CHOSEN?**

You have met the inclusion criteria for this study:

- 1. Age at recruitment Individuals 18 years or older;
  2. Currently a medical student in Klang Valley, Selangor and living in Malaysia;
  3. Willing and able to provide written informed consent;
  4. Being able to read and understand English;
  5. Have reliable Internet access at home or at university;
  6. Proficiency (self-reported) in basic computer/internet skills (as required to participate in the RCT and complete online assessments, etc.).

**WHO SHOULD NOT ENTER THE STUDY?**

The esclusion criteria for this study are:

- 1. Evidence of a sleep disorder (e.g., possible obstructive sleep apnoea, restless legs syndrome;
  2. Currently consumes alcohol;
  3. Medical history contraindicating use of CBT-I, for example, (a) epilepsy (self-report) within the preceding

12 months, or (b) recent cardiac surgery, or (c) currently in an attack phase of multiple sclerosis;

- 1. Individuals whose work schedule includes night shifts;
  2. Pregnancy;
  3. Inadequate opportunity to sleep or living circumstances that prevent modification of sleep pattern (e.g., having an infant residing at home);
  4. Currently receiving psychological treatment for insomnia;
  5. Registered at or under the care of any of the trial centres;
  6. Serious physical health concerns necessitating surgery or with a prognosis of under 6 months;
  7. Those taking prescribed sleeping pills more than 2 nights in the past 2 weeks prior to study entry; and
  8. Those with suicidal ideation with intent. This study will not omit participants for any other physical or mental health problems.

**WHAT WILL BE THE BENEFITS OF THE STUDY:**

**(a) TO YOU AS THE PARTICIPANTS?**

If you choose to participate in this study, you may gain several benefits such as:

- - 1. Improve in sleep quality by learning techniques to identify and modify the negative thoughts and learning how to reduce presleep arousal.
    2. Improve sleep hygiene by learning specific skills to cope with difficulties initiating sleep or maintianing good sleep.
    3. Increase number of social support as the intervention will be run in a group setting.
    4. Free consultation of the intervention from experienced therapist.

**(b) TO THE INVESTIGATOR?**

- - 1. Able to train the respondents with poor sleep quality on cognitive techniques, behavioral strategies, and sleep hygiene in order to improve sleep quality.
    2. Able to improve the quality of sleep related rehabilitaion in medical univesity in the country.
    3. Able to promote the support system among respondents in order to reduce dependency on profecctional experts.
    4. Obtain data and information on te efcctiveness of the intervention from Malaysia to be compared with other countries in the form of publications and intrenational conferences.

**ARE THERE ANY RISKS?**

Similiar studies have been conducted in overseas previously. As far as it is concerned, the is almost no or minimal risk should be anticipated by the respondent. Instead you may feel more confident in dealing with your sleep and you life. However, if you feel you will be exposed to psychological related risk (e.g., feeling embrassed, worried, or upset) and /or socially related risk (e.g., loss of privacy and reputations), please share it with the researchers before, during and /or after the study. It is important howerver to note that the therapy will be conducted professionally by an expert therapist who have experienced handling group therapy. Rules and regulations of cinducting group therapy will also be imposed in order to minimise the risks, if any.

**ARE THE POSSIBLE DRAWBACKS?**

Intervention (a) and (b) are psychotherapies whereby it involves discussion between the therapist and the respondents. If there are any side effects from medications taken, respondents may seek advices from their respected physician.Whatbis importants in this study, professional supervisions by professional experts will help both the therapist and respondents from violating any ethical problems.

**WILL THE INFORMATION AND MY IDENTITY REMAIN CONFIDENTIAL?**

Definitely. All data in this study will remain confidential.The analyst will use the ID number and only the raw scores will be mentioned without revealing the identity of each participant.

**WHO SHOULD I CONTACT IF I HAVE ADDITIONAL QUESTIONS DURING THE COURSE OF THE RESEARCH?**

Should you have any quiries regarding the study, please do not hesitate to contact the following person that are

responsible in this study.

Dr. Firdaus Binti Mukhtar

Professor and Clinical Psychologist

Faculty of Medicine & Health Science

Department of Psychiatry

Universiti Putra Malaysia

43400 Serdang

Selangor

Tel: 012-302 6353

Email: drfirdaus@upm.edu.my Vijandran A Mariappan

Researcher/ PhD student

Faculty of Medicine & Health Science

Department of Psychiatry

Universiti Putra Malaysia

43400 Serdang

Selangor

Tel: 016-2210132

Email: gs64623@student.upm.edu.my
