# Supplementary material for: Effects of Internet Cognitive Behavioral Therapy for Insomnia and Internet Sleep Hygiene Education on Sleep Quality and Executive Function Among Medical Students in Malaysia: Protocol for a Randomized Controlled Trial
Source: JMIR Res Protoc. 2024 Dec 11;13:e59288. doi: 10.2196/59288 (PMC11669887; doi:10.2196/59288)
Supplement: Multimedia Appendix 4 [file resprot_v13i1e59288_app4.docx]

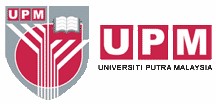
**JAWATANKUASA ETIKA UNIVERSITI UNTUK PENYELIDIKAN**

**MELIBATKAN MANUSIA (JKEUPM)**

**UNIVERSITI PUTRA MALAYSIA, 43400 UPM SERDANG,**

**SELANGOR, MALAYSIA**

# CONSENT FORM (RESPONDENT)

**RESEARCH TITLE :**

The Effectiveness of Group Internet Cognitive Behavior Therapy for Insomnia for Medical Students

**RESEARCHERS:** Dr. Firdaus Binti Mukhtar & Vijandran A Mariappan

I……………………………………............................. Identity Card No....................................................... address………………………………………………………………………………………………………..............................

.........................…………………………………..hereby voluntarily agree to take part in the clinical research *****(clinical study using questionnaire) as specified above.

I have been informed about the nature of the clinical research in terms of methodology, possible adverse effects and complications (refer to Respondent’s Information Sheet). I understand that I have the right to withdraw from this clinical research at any time without giving any reason whatsoever. I also understand that this study is confidential and all information provided with regard to my identity will remain private and confidential.

I wish to *****know / don’t wish to know the results of the tests performed on my sample.

*** delete where necessary**

Signature ……..………………………… Signature ……..………………………….

(Respondent) (Witness)

Date :………………………………….….. Name :………………………………….…..

I/C No. :………………………………….…..

I confirm that I have explained to the respondent the nature and purpose of the above-mentioned clinical research.

Date ……..………………………… Signature ……..………………………….

(Researcher)
